# Structure-based design of gRNA for Cas13

**DOI:** 10.1038/s41598-020-68459-4

**Published:** 2020-07-14

**Authors:** Srinivas Bandaru, Mika Higashide Tsuji, Yurika Shimizu, Kaya Usami, Suni Lee, Naoko Kumagai Takei, Kei Yoshitome, Yasumitsu Nishimura, Takemi Otsuki, Tatsuo Ito

**Affiliations:** 1 Department of Hygiene, Kawasaki Medical University, 577 Matsushima, Kurashiki, Okayama 701-0192 Japan; 2 Department of Applied Chemistry and Biotechnology, Faculty of Engineering, Graduate School of Engineering, University of Fukui, Bunkyo 3-9-1, Fukui, Fukui 910-8507 Japan; 3 Department of Pathophysiology, Periodontal Science, Okayama University Graduate School of Medicine, Dentistry, and Pharmaceutical Sciences, Okayama, Okayama 700-8558 Japan; 4 Okayama University Medical School, Okayama, 700-8558 Japan

**Keywords:** Biological techniques, Genetic association study, Sequencing

## Abstract

Cas13 endonuclease activity depends on the RNA local secondary structure with strong preference for single-stranded (SS) regions. Hence**,** it becomes indispensable to identify the SS regions for effective Cas13 mediated RNA knockdown. We herein present rational gRNA design by integrating experimental structure-seq data and predicted structural models. Utilizing structure-seq data for *XIST* transcript, we observed that gRNAs targeting the SS regions significantly induce transcript knockdown and cleavage than those targeting double-stranded (DS) regions. Further, we identified the “central seed region” in the gRNA that upon targeting the SS regions efficiently facilitates Cas13 mediated cleavage. In our following pursuits, we considered the scenario wherein experimental structure-seq data is not available, hence we used *SS18-SSX2* fusion transcript indicated in synovial sarcomas and computationally predicted its structure. We observed that gRNAs targeting the SS regions predicted from the structure, efficiently induced necrosis compared to gRNAs that target the DS regions. In conclusion, for the effective RNA knockdown, the Cas13 mediated targeting strategy presented herein emphasizes the designing of gRNAs specifically targeting SS regions by utilizing structural information. Further, this strategy, in turn, can be anticipated to narrow the search space for gRNA design (by exclusively targeting SS regions) especially when lncRNAs are the targets.

## Introduction

Bacterial CRISPR-Cas adaptive immunity system has emerged as one of the most robust and precise genetic manipulation tools ever discovered in the field of molecular life sciences^1,2^. Recent pursuits in large-scale data mining of the microbial genomes led to the identification of unexplored classes of RNA-targeting CRISPR-Cas systems including C2c1, C2c2, and C2c3. These systems comprise of unique interference module distinct from previously characterized DNA targeting systems^3^. Of particular importance, C2c2—designated as Cas13—has been extensively repurposed in transcriptome engineering for its programmable possibilities in RNA knockdown and RNA editing, transcript tracking, and disease diagnosis^4^.

Unlike C2c1and C2c3 endonucleases, Cas13 exclusively comprises of two enzymatically active Higher Eukaryotes and Prokaryotes Nucleotide-binding (HEPN) RNase domains^5^, which induce *cis*- and *trans* RNA cleavage via crRNA-guided effector complex (crRNA-Cas13)^6^.

Recent efforts to assess the influence of RNA secondary structure on Cas13 efficiency in bacterial and mammalian cells have shown its strong cleavage preference for ssRNAs, while being target inaccessible to higher-order structures^4^^.^ In addition, preference for ssRNA transcripts is established from the studies which suggest that the spatial distribution for Cas13d in bacterial screens have strongly depleted target sites at the SS regions alone^7^.

In sheer contrast to Cas9 endonucleases, the recruitment of crRNA-Cas13 effector to the target RNA does not induce local melting necessary for strand separation, thereby hampering crRNA binding to the DS regions^8^. This rationalizes why the knockdown efficiency of Cas13 is explicit for SS regions but not for substantially base-paired DS regions.

Since Cas13 selectively prefers single-stranded regions in RNA, we herein present a structure-based gRNA design strategy for exclusively targeting the SS regions by utilizing structural information derived from structure-seq data and structure prediction methods.

## Results

### crRNA-Cas13 mediated transcript knockdown utilizing structure-seq information

In the present investigation, we used the *XIST* transcript for two main reasons. First, *XIST* is long non-coding RNA with a transcript length of 19,245 base pairs, so the likelihood of having single- (loops) and double-stranded regions (stems) was higher than for RNAs with shorter lengths. Second, the availability of a curated structure-seq profile with comprehensive structural information made the *XIST* transcript a potential candidate for testing our hypothesis.

**Figure 1:**
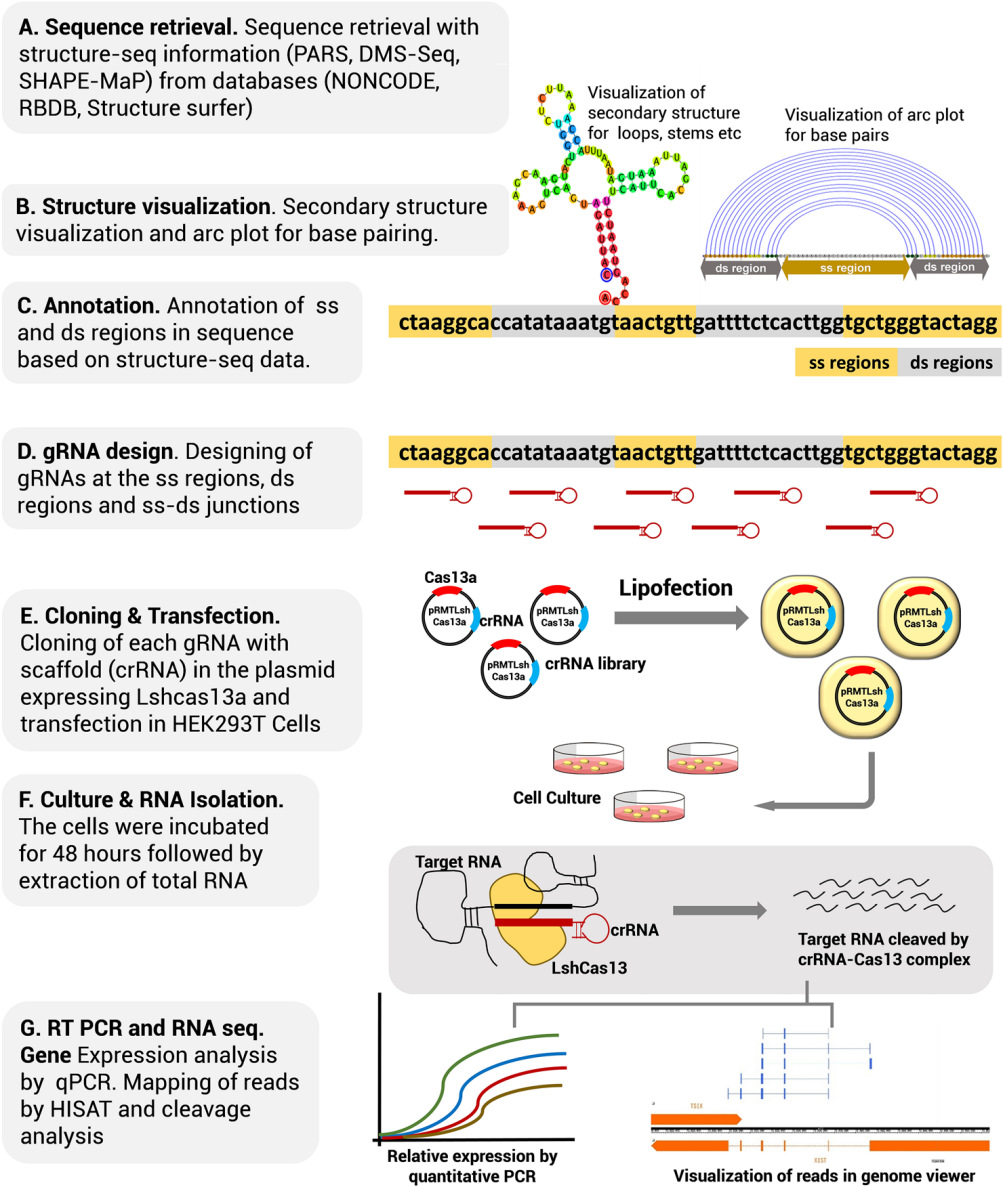
Schematic overview of the experimental design.

The *XIST* sequence along with its structure probing information was retrieved from the NONCODE database (PARS probing experiments; Dataset source: PARS(V1_Child-S1_Child))^9^. RNA secondary structures were visualized in RNAstructure v 6.0.1 suite. The program was selected for its ability to constrain or restrain structure based on structure-seq probing data. Followed by the mapping and annotating the structural details, gRNAs were designed to target the SS regions (loops), the DS regions (stems) and the junctions of SS-DS regions (stem-loop junctions). The plasmid library was constructed by cloning crRNAs in the pRMT vector containing human optimized LshCas13a insert, followed by transfection in the HEK293T cells and transcript quantification by exon specific primers. The complete workflow schema is demonstrated in Fig. 1.

**Figure 2:**
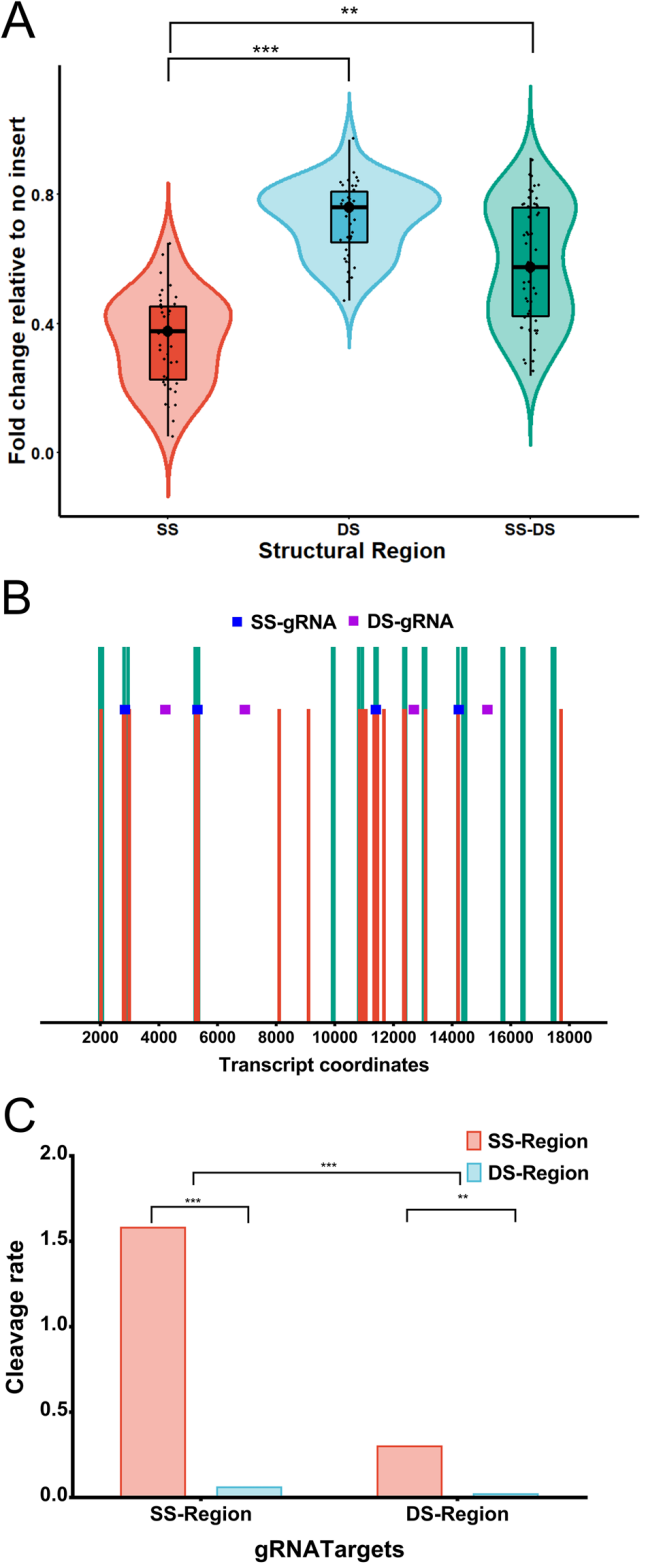
*XIST* knockdown upon gRNA targeting the SS and DS regions in the transcript. (**A**) Set of gRNAs targeting the SS regions (loops) brings about a significant knockdown of *XIST* transcript compared to the ones targeting the DS regions (stems). gRNAs targeting the SS-DS regions (stem-loop junctions), however, demonstrate wide expression range having few gRNAs efficient for transcript knockdown. (**B**) The red vertical bars indicate the cleaved locus in the transcript as analysed from RNA-seq and HISAT 2.0 mapping. Green vertical bars indicate SS regions; annotated in reference to the structure-seq data. A significant overlapping of the cleaved nucleotides onto the SS regions shows the selective cleavage preference of Cas13 endonucleases for SS regions. (SS-gRNA and DS-gRNA respectively target SS and DS regions) (**C**) Cleavage rate in an RNA-seq experiment was determined by the ratio of cleaved nucleotides in a given transcript region (SS or DS) to the uncleaved nucleotides. Note that the cleavage rate is significantly high for SS regions compared to DS regions. Although certain gRNAs directed for SS regions show a minor and unspecific cleavage at DS regions and vice versa, nevertheless their number was insignificant compared to gRNAs those demonstrating strand-specific cleavage.

We observed that the expression of *XIST* was diminished by almost five-fold upon the targeting of gRNAs to the SS regions in the transcript in comparison to those targeting DS regions (p < 0.001), implying that the gRNAs targeting DS regions to a large extent were ineffective in knocking down the transcript (Fig. 2A, Supplementary information Table 1). To validate the credibility of expression results, RNA-seq was performed to identify the possible cleavage in the *XIST *transcript induced by crRNA-Cas13 effector. A close perusal of the aligned segments onto the SS and DS regions revealed an extensive cleavage at the SS regions in the transcript upon targeting gRNAs at the SS regions (Fig. 2B). The cleavage rate on SS regions was remarkable; it was several fold higher than on the DS regions (Supplementary information-1, Table 2). Although certain gRNAs target specifically SS regions or DS regions, other gRNAs brought about cleavage at both SS and DS regions (probably due to collateral cleavage of activated Cas13), nevertheless, the gRNAs targeting the SS regions was far more efficient to induce cleavage at the SS region than at DS regions (p < 0.001). On the other hand, gRNAs targeting DS regions were poor in inducing cleavage either at SS or DS regions (Fig. 2C). This analysis thus shows that SS regions can serve as cleavage hot spots for Cas13 endonuclease activity and that gRNA design directed for SS regions forms an efficient stratagem for Cas13 mediated RNA knockdown. In fact, the present observation is in coherence to the previous studies demonstrating strong preference of crRNA-Cas13 effectors for SS region^4^. Therefore, for efficient knockdown of the transcript, it is essential to target the SS regions relying on structural information derived from structure-seq data.

### Central seed binding region in gRNA crucial for transcript cleavage.

**Figure 3:**
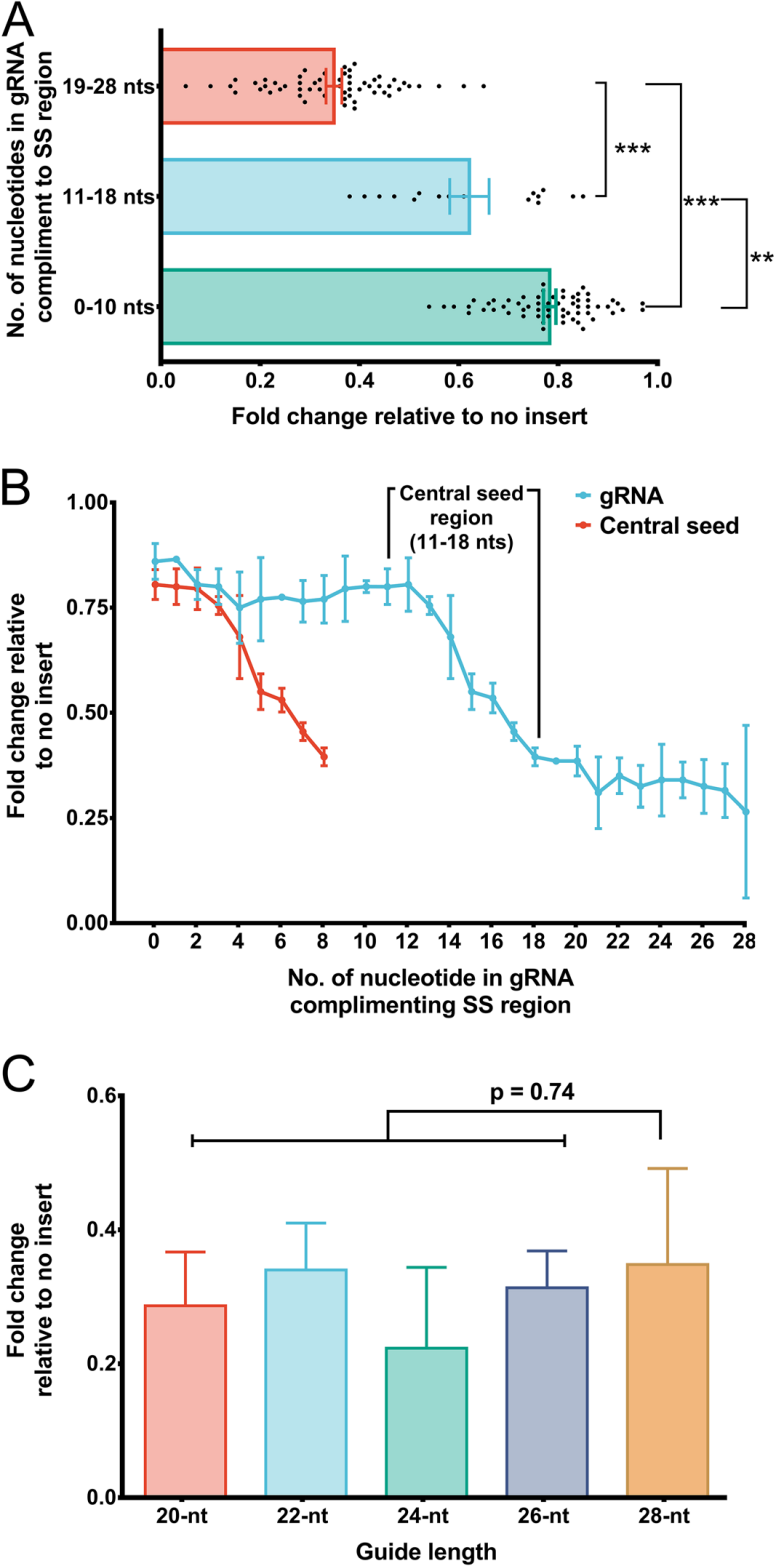
Requirement of single-stranded nucleotides in the transcript for Cas13 activity. (**A**) 28 nt gRNAs stratified into 3 subsets depending upon their complementarity to nucleotides in the SS region. Among the three subsets, gRNAs with 0–10 nts complementing nucleotides of SS regions bring about poor knockdown of the transcript which is evident from high expression values. For the subset of gRNAs that complement SS region nucleotides with 11–18 nts, a wide range of transcript expression values was observed. This subset comprised only a few gRNAs that induced efficient knockdown (gRNAs which complemented SS regions nucleotides with 17–18 nts). The subset of gRNAs complementing the SS regions with 19–28 nts was the most efficient among the three subsets to induce knockdown. (**B**) The extent of transcript knockdown analyzed in relation to the number of each nucleotide in guide RNA that complement the nucleotides in SS regions in SS-DS junctions. gRNAs with 0–10 nts complementing SS regions bring about poor knockdown, however, transcript expression declines with gRNAs those complementing nucleotides of SS regions starting with 11 nts reaching up to 18 nts. While, gRNAs with more than (and including) 18 nts (18–28 nts) complementing SS region nucleotides demonstrated superior knockdown of the transcript. It is, in fact the, the “central seed” binding region in the gRNA with 8 central bases (11–18 nts) which upon complementing SS region in the transcript brings about significant knockdown. Moreover, when complementarity exceeds the central seed bases, there was no apparent change in the transcript knockdown. This implies that, the central seed region in the gRNAs upon complementing SS regions is sufficient to promote Cas13 mediated transcript knockdown. (**C**) An insignificant change in the *XIST* expression upon targeting with 20–28 nts long gRNAs shows that Cas13 mediated transcript knockdown is tolerated by varying gRNA lengths, provided their central seed bases target the SS regions of the transcript.

**Figure 4:**
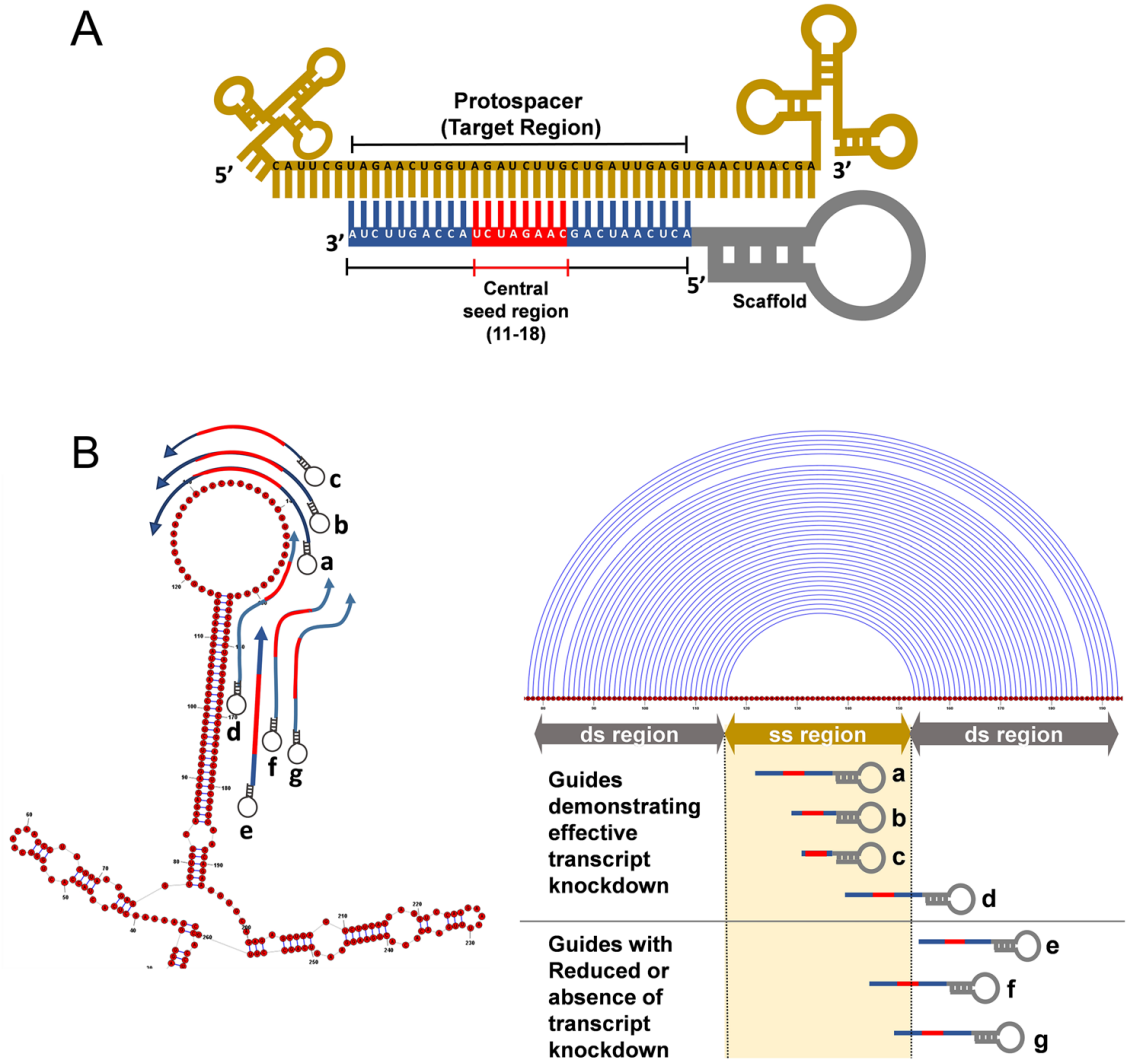
Binding of central seed region in gRNA crucial for Cas13 mediated cleavage. (**A**) Schematic of central seed region of the guide in the crRNA binding the protospacer (target) region in the RNA. (**B**) (Left) Schematic illustrating gRNAs targeting the SS and DS regions in the RNA structure. The central segment (in Red) in the gRNA is the central seed region. (Right) SS and DS stranded regions in the RNA structure are shown as arc plot, along with the targeting gRNAs. gRNAs (a, b, c & d) of varying length induces Cas13 mediated transcript knockdown as their respective central seed complement the SS regions in the RNA. gRNA (e) with its entire nucleotides targeting DS regions and gRNAs (f & g) with its central seed region respectively complementing SS-DS and DS regions in the transcript show reduced or absence of knockdown.

Our further motive was to evaluate the requirement of the single-stranded nucleotides in the target RNA necessary to complement gRNA in order to facilitate efficient Cas13 cleavage. Hence, we designed a total of 54 gRNAs of 28 nts in length that complement SS-DS regions (stem-loop junctions) with its varying number of bases to the given region, and we evaluated these gRNAs for their ability to facilitate transcript knockdown. It was interesting to note that a poor knockdown was observed when gRNAs complemented the SS regions with less than 10 nts in contrast, gRNAs complementing the SS regions with 18 or more nts induced more efficient knockdown (Fig. 3A, Supplementary information-1, Table 3). Further, a steep decline in the transcript expression was apparent for gRNAs complementing 11–18 nt in SS regions (Fig. 3B). This analysis convinces the presence of a putative “central seed” binding region in the gRNA with 8 central bases (from 11–18 nts) (Fig. 4A). A close perusal showed that the complementation of all the 8 bases of the central seed regions in gRNA to SS region was adequate to induce transcript knockdown (irrespective of terminal nucleotides in gRNA complementing DS regions) (Figs. 3B, 4B, Supplementary information-1, Table 4).

Next, we designed gRNAs with varying lengths of 20–26 nt long. We did not find significant change in *XIST* expression with varying the lengths of gRNAs (Fig. 3C, Supplementary information-1, Table 5). This finding suggests that gRNAs with lengths ranging from 20–28 nt are tolerated for Cas13-mediated knockdown, provided that the central region is retained (Fig. 4B). This observation is in agreement with the findings of Liu et al.^10^ and Tambe et al.^11^, which also emphasized base pairing of central “binding seed” region of gRNA with the target RNA as an absolute necessity to induce Cas13-mediated cleavage. These binding seed regions was more important than its terminal nucleotides.

### Protospacer flanking sites (PFS) and Cas13 cleavage

**Figure 5:**
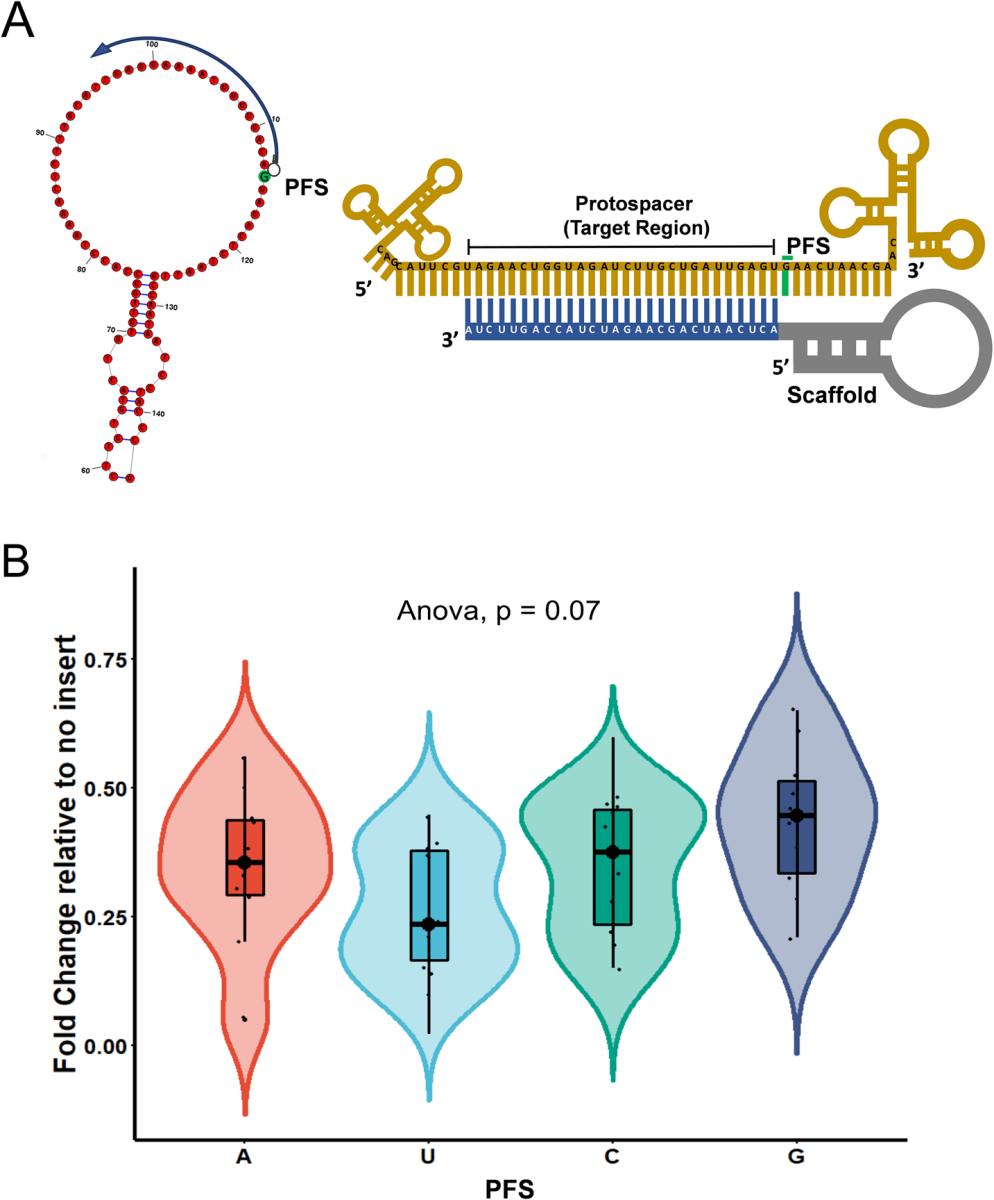
Protospacer flanking sequence (PFS) for Cas13 mediated RNA targeting. (**A**) Schematic representation for gRNAs targeting SS regions with PFS (nucleotide in green background) in the RNA structure. Corresponding to the structure, illustration shown with gRNA aligning to the target region with a given PFS (green bar). (**B**) *XIST* Transcript expression shows insignificant PFS restrictions for LshCas13a in the HEK293T cells.

To evaluate the preference of LshCas13a for protospacer flanking site (PFS) in the SS regions of the *XIST*, we designed PFS compatible guides flanking the protospacer sequence (Fig. 5A). The expression analysis confirmed insignificant PFS restrictions of LshCas13a in the HEK293T cells (p = 0.07) (Fig. 5B, Supplementary information-1, Table 6). Albeit insignificant, gRNAs targeting at the G-PFS resulted in slightly less transcript knockdown. The PFS based rules in mammalian cells have always been contradicting, which can be due to the selection of Cas13 orthologs or even cell type, such discrepancies nevertheless demand further investigations.

### Pseudoknots do not influence Cas13 mediated cleavage

**Figure 6:**
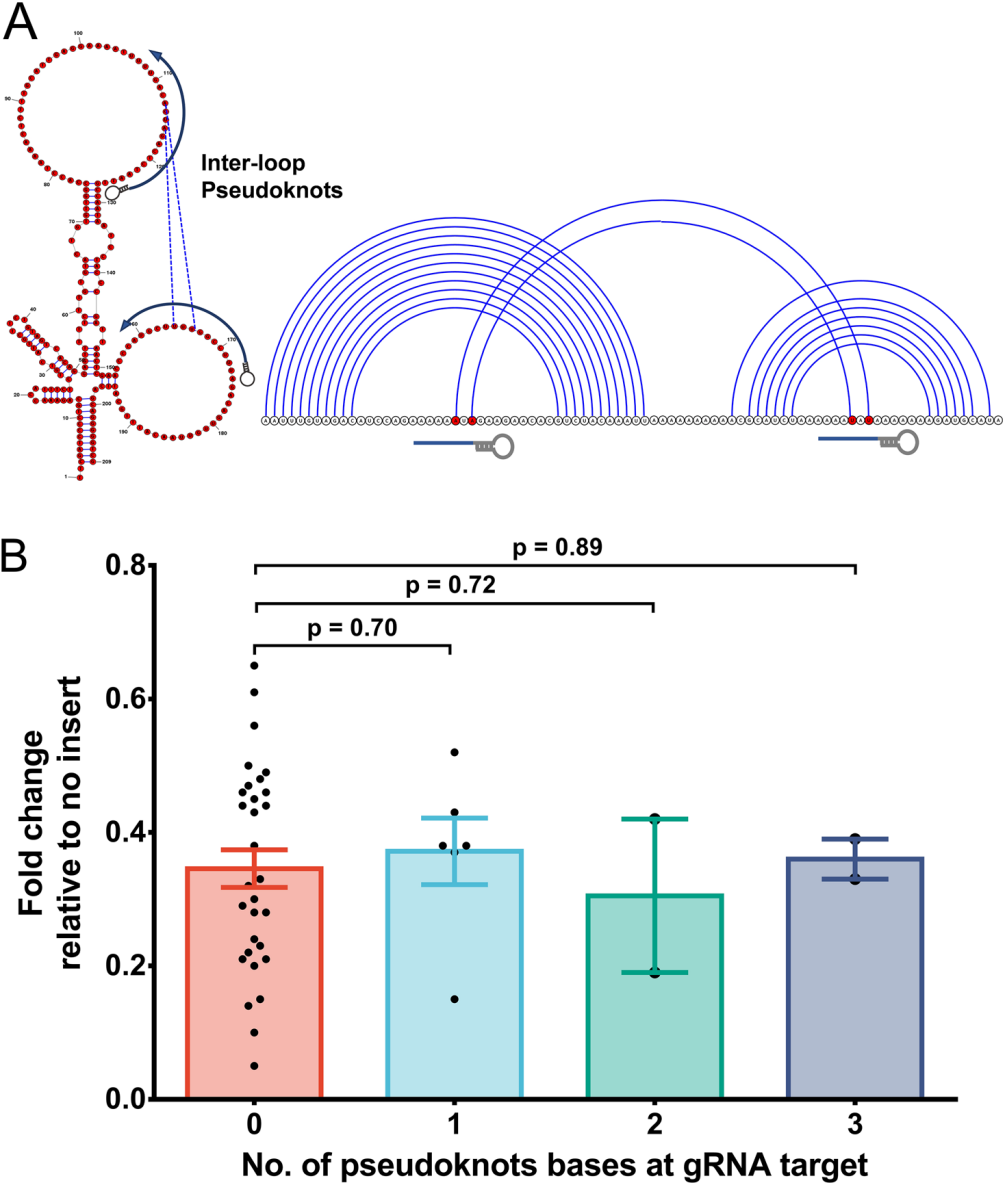
Pseudoknots do not influence Cas13 mediated transcript knockdown. (**A**) (Left) Schematic representation of RNA structure with gRNAs targeting the bases participating in inter-loop pseudoknot formation. (Right) Corresponding to the structure, shown is the arc plot representation of the pseudoknots with gRNA targets. (**B**) Irrespective of the number of pseudoknots participating bases at the gRNA target location, there was an insignificant change in the *XIST* expression.

Pseudoknots are structurally diverse groups in the RNAs characterized by the hydrogen bond interactions of single-stranded loops to the adjacent stems or loops (Fig. 6A). Because Cas13 prefers to cleave single-stranded regions, we presumed that pseudoknot bonding may hamper Cas13 mediated cleavage. To test this, we computationally predicted the pseudoknots in the *XIST* transcript by an integer programming based (IP) IPknot package^12^ and targeted pseudoknoted regions by gRNAs. Interestingly, there was an insignificant change in the transcript knockdown upon targeting of gRNAs onto pseudoknots-enriched loops compared to those lacking them (Fig. 6B, Supplementary information-1, Table 7). The exact explanation for this observation is elusive; however, one possible explanation may be that the long-range H-bonds in pseudoknots have relatively weaker interactions than the anti-parallel base-pairing in the stem region, which likely facilitates unobstructed crRNA-Cas13 binding to the single-stranded loops.

### crRNA-Cas13 mediated knockdown of transcript on predicted RNA structure

**Figure 7:**
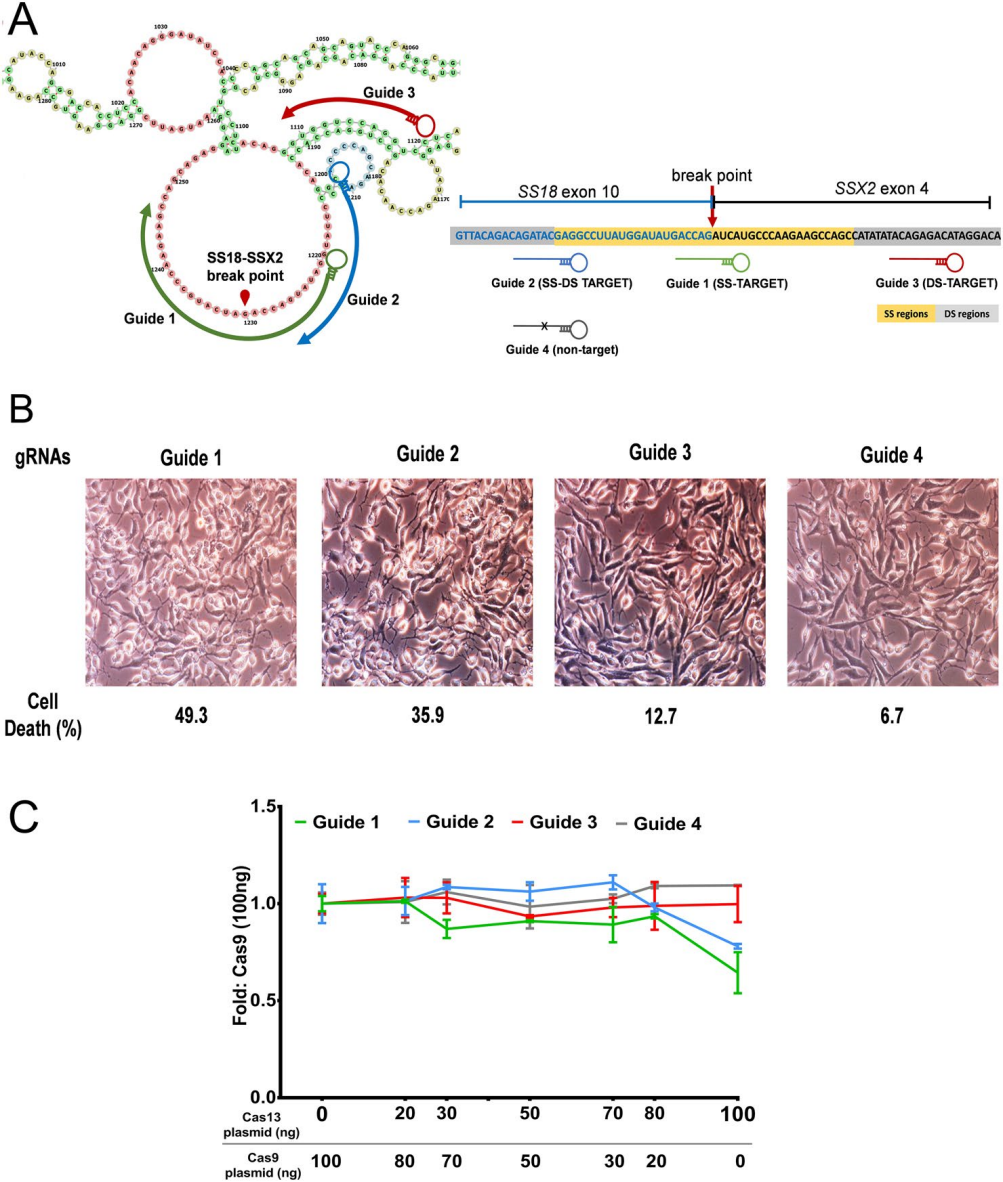
Targeting of SS18-SSX2 fusion transcript. (**A**) Schematic showing predicted structure of SS18-SSX2 fusion transcript with gRNA targeting at SS regions (Guide 1), DS regions (Guide 2), and SS-DS regions (Guide 3) of the transcript, along with the gRNA having no target complement to transcript (Guide 4). (**B**) Cell viability by trypan blue staining. gRNA-Cas13 targeting SS18-SSX2 breakpoint present in the SS regions induces efficient cell death in SYO-1 cells compared to the gRNAs those targeting the DS regions or SS-DS junctions. (**C**) SYO-I cell viability correlates with Cas13 expression. Both Cas13 and Cas9 expressing plasmids were transfected into SYO-I cells with crRNAs. Viability of SYO-1 cells having gRNA targeting SS18-SSX2 significantly decreased upon increasing the concentration of Cas13 especially for Guide 1 that targets the SS region of the transcript. The cell viability was however independent of Cas9 concentration. The total amount of induced plasmid was fixed at 100 ng/ well. Cell viability was assessed using WST-1 Cell Viability Assays (Sigma-Aldrich).

Next, we considered for structure-based knockdown by crRNA-Cas13 of the RNA for which structure-seq data is unavailable. We used the SS18-SSX2 translocated fusion transcript t(X;18)(p11;q11), indicated in biphasic synovial sarcomas^13^. Previous studies have shown that the expression of SS18-SSX2 protein promotes the survival of synovial sarcoma cells (SYO-1)^14^. We therefore, presumed that the annihilation of the SS18-SSX2 fusion transcript by Cas13 may induce necrosis, which in turn can be evaluated by cell viability assays. We computationally predicted the SS18-SSX2 fusion structure using RNAstructure software package, which includes several folding algorithms including the dynamic programming algorithm^15^ and nearest neighbour parameters^16^. gRNAs were designed based on the predicted SS regions (loop regions) and DS regions (stems) (Fig. 7A). Interestingly, trypan blue staining showed that the SYO-1 cells with gRNA targeting the SS regions (loops) of SS18-SSX2 were significantly necrotized in comparison to cells having gRNAs with the DS region target (Fig. 7B). To ensure that Cas13-mediated cleavage of the fusion transcript induced necrosis of cells, SYO-1 cells were transfected with Cas13 as well as Cas9 plasmids. Interestingly, viability of SYO-1 cells significantly decreased upon increasing the concentration of Cas13 especially for the gRNA that targets SS regions of the transcript, while the viability was independent of Cas9 concentration (Fig. 7C). These results, thus, emphasize the importance of structure-based RNA targeting by Cas13 endonucleases to induce cell death, which may become a prospective therapeutic strategy for targeting cancer cell and types. Overall, this investigation serves as a complement to the aforementioned *XIST* transcript targeting, which proves that efficient knockdown of the transcript can be brought about by targeting gRNAs at the SS regions.

## Discussion

CRISPR Cas13 system has emerged as one of the most powerful RNA engineering toolboxes which have a startling array of applications ranging from programmable RNA knockdown, editing (REPAIR^17^, RESCUE^18^), nucleic acid detection (SHERLOCK^19^ and viral detection CARVER^20^). CRISPR-Cas13 opens the next-generation molecular diagnostics and therapeutics.

The selective preference of Cas13 for single-stranded regions in RNA can be attributed to its inability to assist crRNA interrogation at the double-stranded regions. In contrast to crRNA-Cas9 effector, crRNA-Cas13 effector fails to trigger local melting in the RNA duplex. As a result, the binding of crRNA to its complementary sequence by displacing the opposite strand merely remains unsuccessful^21^. It remains if Cas13 is unable to initiate melting or if thermodynamics of local RNA motifs forbids strand displacement. In either case, the inaccessibility of crRNA to the DS regions makes Cas13 to prefer single-stranded regions for cleavage. Indeed, for this reason, and asserting to the previous studies^4,8,10^, we found Cas13 mediated cleavage extensively at the single-stranded regions, this is in agreement with previous studies. The results from our analysis suggest that a structure-based targeting of RNA is extremely important for gRNA design for Cas13 RNA cleavage.

Our study supports the existence of a “central seed region” as proposed in the previous studies^10,11^, which reported the absolute necessity of the central bases of a gRNA for Cas13 mediated RNA cleavage. The importance of the central seed region becomes more obvious from structural insights of crRNA-RNA interactions. These show that the central seed regions have solvent-exposed sugar-phosphate backbone that facilitate Watson–Crick base pairing with the target RNA while being close to the HEPN catalytic domain^8,10,11^. Therefore, we herein show that for effective target cleavage, the central seed region of the gRNA must mandatorily complement the single-stranded region, regardless of its length or its terminal nucleotides being complementary to the DS regions.

Unlike for Cas9, PFS dependency rules for Cas13 nucleases are less strict and vary across orthologs, thus making it a flexible targeting system. For example, 3′ H (non G) PFS restrictions were shown for *Leptotrichia shahii* (LshCas13a) in MS2 immunized *E. Coli* strains^22^. In contrast, *Eubacterium siraeum* (EsCas13d) and* Ruminococcus sp* (RspCas13d) completely lacked PFS requirements in in vitro or in vivo bacterial screens^7^. In mammalian cells however, the results have been contradictory, *Leptotrichia wadei* (*LwaCas13a*) demonstrated PFS requirements, while *Leptotrichia shahii* (LshCas13a) did not^23^. In our experiments using LshCas13a in HEK293T cells, although significant PFS requirement was not observed, nevertheless, there was a marginal bias for the requirement of 3′G PFS. The PFS requirements for Cas systems still need extensive study which may add more sequence-based rules across the nuclease classes and types.

In recent years, high-throughput next-generation sequencing with a wide selection of probing methods (PARS, DMS, icSHAPE, Mod-Seq) led to structure-seq data publicly available for various genomes involving diverse cell types, solvent effects, and epigenetic influences. For example, repositories like RMDB (RNA Mapping Database) currently houses staggering 16,897,456 data points obtained from 1,164 structure-seq experiments with 714 entries on 143,155 constructs^24^. Likewise, Structure Surfer database which contains transcript-wise indexing of probing data from Human, Mouse, and Arabidopsis genomes also serves as a trusted repository for retrieving structural information^25^.

In the majority of cases, structural-seq probing information may be unavailable for the RNA in question. In such circumstances, computational structure prediction stands as a second choice. In this view, we predicted the structure of the SS18-SSX2 fusion transcript indicated in synovial sarcomas because of the unavailability of structure data for fusion transcripts. Programs like the ViennaRNA package^26^, RNAstructure^27^, and Mfold^28^, which incorporate dynamic programming algorithms have been efficient in creating the most probabilistic models of RNA secondary structures close to those in vivo. However, it is useful to predict RNA secondary structure by complementary algorithms, as no program completely automates the structural states in the cellular milieu. Considering the minor fallacies of computational prediction, gRNA design relying on predicted structures forms only a substitute for structure-seq data, and is, not a replacement for it.

To date, structure-seq experiments that resolve the structure at a single- nucleotide happen to be the most reliable source of in vivo RNA secondary structure. In light of a strong preference of Cas13 for single-stranded regions, it becomes indispensable to identify the structural motifs, especially while targeting lncRNAs. It can be extremely daunting to design tens and thousands of gRNAs targeting the entire length of lncRNAs and to test for transcript cleavage. Moreover, most of the regions in lncRNAs from double-stranded or higher-order structures, so the probability that several gRNAs target SS regions can be considerably low. Therefore, the ambiguous practice of gRNA design in lack of structural information finally amounts to the labour-intensive experimentation with numerous trials that consume time and resources. We address this concern by the rational design of gRNAs based on RNA structure information (Step-wise workflow for structure-based gRNA design is provided in Supplementary information 1, Fig. 2). The method of gRNA design presented herein is a knowledge-based strategy that requires design of a handful of gRNAs that cut both experimental efforts and costs, as could not otherwise be anticipated from the ambiguous targeting of RNA in the absence of structural information.

## Conclusion

The structure-based strategy introduced herein can be anticipated to narrow the search space for gRNA design and thus, limit the ambiguous targeting along the entire length of the transcript. In addition, since this strategy integrates structural information, identifying single-stranded region from such information and designing corresponding gRNAs reasonably increases the likelihood of transcript knockdown, as would not be expected in the absence of structural information.

## Materials and methods

### Transcript selection and retrieval

The transcript sequence of *XIST* (ENSEMBL ID ENST00000429829.6) with structural information deduced from Parallel Analysis of RNA Structure (PARS-seq) probing (Dataset source: PARS(V1_Child-S1_Child), was retrieved from NONCODE database (NONCODE ID: NONHSAT137541.2).

### RNA modelling and visualization

RNA modelling and base pair probability prediction were performed with Mathew Lab’s RNAstructure, Structure Editor Version 1.0 suite (https://rna.urmc.rochester.edu/RNAstructure.html) ^15^. Briefly, the sequence with structure-seq data was modelled with a maximum percentage difference of 10 kcal/mol using dynamic programming algorithms embedded in the suite. The modelled structures were saved in .ct files for further secondary structure analysis. The single and double-stranded regions were visualized as an arc plot that was colour coded with partition functions for each nucleotide. Forna structure viewer (https://rna.tbi.univie.ac.at/forna/) ^29^ was used for interactive visualization of the secondary structures.

### gRNA design

gRNAs were designed in the SnapGene Viewer Version 4.1.9 (https://www.snapgene.com/). The gRNAs were further validated for its target specificity by similarity search using GGGenome Blast (https://gggenome.dbcls.jp/) against total human genome. Additionally, gRNAs with self-complementarity and propensity to form dimers were not considered. Prior to oligosynthesis, BbsI restriction sequence was added at the 5′end of the designed gRNAs. All the gRNA oligonucleotides were procured from Integrated DNA Technologies, Inc. (IDT) (Coralville, IA). The details of gRNAs designed for the study are provided as a supplement (Supplementary information-2, sheet 2 (gRNAs for *XIST*) &4 (gRNAs for *SS18-SSX2*)).

### Generation of Cas13 mammalian expressing vector pRMT.Lsh and cloning of crRNAs

pX458 (Addgene: Plasmid #48138) vector was modified by replacing a sgRNA locus into a gRNA sequence with two BbsI sites, and Cas9 locus was replaced by Lsh cDNA from pZ003 (Addgene: LshC2c2 locus) using Gibson Assembly protocol (https://www.addgene.org/protocols/gibson-assembly/). crRNA libraries were constructed by cloning each crRNA in the vector by standard gene cloning protocols. All the pRMT1 plasmids harbouring gRNA of interest were isolated and purified using QIAGEN QIAprep Spin Miniprep plasmid isolation kit and were confirmed for inserts by Sanger sequencing.

### Cell culture and transfection

HEK293T cells were procured from ATCC (ATCC CRL-3216) and maintained in DMEM, supplemented with 2 mM glutamine and 10% FBS and antibiotics (penicillin/streptomycin, 0.5 ml) at an atmosphere of 5% CO_2_ and 95% air at 37 °C. The plasmid containing hLshCas13a with crRNA insert was lipofected into HEK293T by Screen*F*ect A *plu*s (https://screenfect.jp/screenfectaplus/) according to the manufacturer’s protocol. The transfected cells were incubated for 48 h in the atmosphere of 5% CO_2_ at 37 °C. Following the incubation, total RNA was collected using trizol method and purified using QIAGEN RNeasy MinElute Cleanup Kit spin columns. SYO-I cells were procured from Okayama University Medical School, Okayama, Japan, which were cultured and transfected with plasmids in a similar way as performed for HEK293T cells.

### Quantitative polymerase chain reaction

RNA was quantified by real-time quantification polymerase chain reaction. 500 ng of total RNA was reverse transcribed with random primers targeting total RNA using a standardized instructions kit. The expression results were expressed as fold relative to the house-keeping internal control GAPDH gene expression. The RT-PCR reaction was performed on StepOne Real-Time PCR Systems (Applied Biosystems). Quantitation, and calling of real-time amplification values were performed on StepOne Software Version 2.2 (https://www.thermofisher.com/). The details of qPCR primers used for the study is provided as a supplement (Supplementary information 2, sheet 3).

### Next-generation sequencing

Nanopore sequencing was performed on MinION device, Oxford Nanopore Technologies (ONT), Oxford, UK (https://nanoporetech.com/products/minion). Sequencing library was generated with 500 ng cDNA using nanopore sequencing Rapid Sequencing Kit (SQK-RAD004) following the manufacturer’s protocol and loaded onto a flow cell (R9.4.1, FLO-MIN106) resting on the MinION device. The MinION device was controlled by ONT’s MinKNOW Version 3.1.8 (https://nanoporetech.com/nanopore-sequencing-data-analysis) software installed on the host computer. Data acquisition, data streaming, and base calling were performed on MinKNOW 1.4.2 operating software. Sequencing run for each library was performed for 48 h. Real-time analytical workflows were provided by EPI2ME Agent Version 2.59.1896509 (https://nanoporetech.com/nanopore-sequencing-data-analysis) of Nanopore technologies. Base-calling files were saved in Nanopore specific file format (.fast5). Complete sequencing was carried out on the computer work station configured with Intel Core 17-7700 K CPU, 4.20 GHz processor with 32 GB RAM running on Windows 10 Pro operating system.

### RNA-seq analysis and data processing

Reads generated from Nanopore sequencing were mapped onto the human genome GRCh38.p12 assembly by HISAT 2.0^30^ on Galaxy v 19.01—an open, web-based platform for biological data analysis (https://usegalaxy.org/). Coverage was calculated at each position and normalized. The mapped reads were visualized by Integrative Genomics Viewer Version 2.5.2 (https://software.broadinstitute.org/software/igv/) ^31^. The complete RNA-seq pipeline designed for the study is provided as a supplement (Supplementary information-1, Fig. 1).

### Cleavage analysis

The cleavage in the transcript brought about by crRNA-Cas13 was determined in comparison to the cells that lacked inserts (control). Cleavage rate in the transcript for a given SS and DS region was determined with the following equation:

$$Rate\,of\,cleavage = \frac{No.\,of\,clevaged\,nucleotides }{{Total\,nucleotides }} \times 100$$

Cleavage locus, number of cleavages and coverage of cleaved fragments in the transcript was analysed from HISAT generated binary alignment mapping files (.bam files). Only reads with mapping quality greater than the Phred-scale value of 30 were used, which corresponds to less than 5% probability that the read is wrongly mapped.

### Statistical analysis

The quantification values of targeted transcripts were expressed as log2 fold relative to the internal control gene. All p values were two-tailed with level of significance values indicated as*p < 0.05, **p < 0.01, ***p < 0.001. The results were expressed as mean ± S.D or median as necessary. All the statistical operations were performed on RStudio Version 3.6.1, and the graphical outputs were generated with GraphPad Prism Version 6.01 (https://www.graphpad.com/).

## Supplementary information

Supplementary file1 (PDF 136 kb)

Supplementary file2 (XLSX 40 kb)
