# Supplementary material for: Structure-based design of gRNA for Cas13
Source: Sci Rep. 2020 Jul 14;10:11610. doi: 10.1038/s41598-020-68459-4 (PMC7360764; doi:10.1038/s41598-020-68459-4)
Supplement: Supplementary file 1 — Supplementary file1 (PDF 136 kb) [file 41598_2020_68459_MOESM1_ESM.pdf]

Supplementary Information - 1

Structure-based design of gRNA for Cas13

Srinivas Bandaru[1], Mika Higashide Tsuji[2], Yurika Shimizu[3], Kaya Usami[4], Suni Lee[1], Naoko Kumagai Takei[1], Kei Yoshitome[1], Yasumitsu Nishimura[1], Takemi Otsuki[1], Tatsuo Ito[1*]

[1]Department of Hygiene, Kawasaki Medical University, 577 Matsushima, Kurashiki, Okayama 701-0192, Japan.

[2]Department of Applied Chemistry and Biotechnology, Faculty of Engineering, Graduate School of Engineering, University of Fukui, Bunkyo 3-9-1, Fukui, Fukui, 910-8507, Japan.

[3]Department of Pathophysiology - Periodontal Science, Okayama University Graduate School of Medicine, Dentistry, and Pharmaceutical Sciences, Okayama, Okayama, 700-8558, Japan.

[4]Okayama University Medical School, Okayama, 700-8558, Japan.

*Author for correspondence

TATSUO ITO (TI)

Mail: tataito@med.kawasaki-m.ac.jp
Call: +81-86-462-1111 (X 27510)
Fax: +81-86-464-1125

*Author for correspondence

TATSUO ITO (TI)

Mail: tataito@med.kawasaki-m.ac.jp
Call: +81-86-462-1111 (X 27510)
Fax: +81-86-464-1125

Table 1. gRNA-Cas13 mediated *XIST* expression upon targeting single and double-stranded regions by 28-nt gRNAs

**gRNA target region (n)**	**Fold change relative to no insert Mean±SD**	**P Value**
Single Stranded (40)	0.78±0.11	
Double Stranded (40)	0.35±0.14	<0.001
SS-DS Junctions (54)	0.59±0.21	<0.01
Anova, p<0.001		

Table 2. Cleavage rate of *XIST* transcript in cells treated with gRNA-Cas13 plasmids relative to cells with no insert

		**No. cleavages (Cleavage rate)**		
		**SS Region**	**DS Region**	**P Value**
**gRNA target region (n)**				
SS Region (4)		16 (1.58)	4(0.02)	<0.001
DS Region (4)		3(0.30)	2(0.01)	
**Cleavage Ratio**				
SS Regions : DS Regions	56.88			
SS-gRNA[a] : DS-gRNA[b]	5.21			
SS-gRNA in SS Regions: SS-gRNA in DS Regions	71.84			
DS-gRNA in SS Regions: DS-gRNA in DS Regions	26.94			

[a]SS-gRNA - gRNA targeting single stranded regions
[b]DS-gRNA - gRNA targeting double stranded regions

Table 3. Expression of *XIST* transcript upon treatment with Cas13 by 28 nt gRNAs stratified into 3 subsets depending upon their complementarity to SS bases in the transcript.

	**Fold change relative to no insert Mean±SD**	**P Value**
0-10 (60)	0.782	
11-18 (16)	0.620	0.072
19-28 (58)	0.348	<0.01
Anova, p<0.001		

Table 4. Expression of *XIST* transcript upon treatment with Cas13 with the number nucleotides in 28nt-gRNA and central seed nucleotides complimenting nucleotides in SS region

**Number of nucleotides in gRNA complimenting nucleotides in SS region (N)**	**Mean ±SD**	**Number of central seed nucleotides in gRNA compliment to nucleotides in SS region (N)**	**Mean ±SD**
0 (40)	0.78±0.11	0 (60)	0.78±0.1
1(2)	0.87±0.01	1(2)	0.8±0.05
2(2)	0.81±0.04	2(2)	0.81±0.06
3(2)	0.8±0.04	3(2)	0.76±0.02
4(2)	0.75±0.08	4(2)	0.68±0.1
5(2)	0.77±0.1	5(2)	0.55±0.04
6(2)	0.78±0.01	6(2)	0.54±0.04
7(2)	0.77±0.05	7(2)	0.46±0.02
8(2)	0.77±0.06	8 (60)	0.35±0.12
9(2)	0.8±0.08		
10(2)	0.8±0.01		
11(2)	0.8±0.05		
12(2)	0.81±0.06		
13(2)	0.76±0.02		
14(2)	0.68±0.1		
15(2)	0.55±0.04		
16(2)	0.54±0.04		
17(2)	0.46±0.02		
18(2)	0.4±0.02		
19(2)	0.39±0.01		
20(2)	0.39±0.04		
21(2)	0.31±0.08		
22(2)	0.35±0.04		
23(2)	0.33±0.05		
24(2)	0.34±0.08		
25(2)	0.34±0.04		
26(2)	0.33±0.06		
27(2)	0.38±0.01		
28 (40)	0.35±0.14		

Table 5. Expression of XIST transcript upon treatment with Cas13 with varying gRNA lengths targeting SS regions

gRNA Mer (N)	Fold change relative to no insert Mean ±SD	P value
28-nt (40)	0.35±0.14	
26-nt (3)	0.31±0.06	0.67
24-nt (3)	0.22±0.12	0.15
22-nt (3)	0.34±0.07	0.92
20-nt (3)	0.29±0.08	0.46
Anova, p = 0.58		

Table 6. Expression of *XIST* transcript with gRNA targets at varying Protospacer Flanking Sequence (PFS)

PFS targeting Guides (N)	Fold change relative to no insert Mean ±SD	P Value
A (10)	0.38±0.15	
U (10)	0.27±0.12	0.18
C (10)	0.35±0.13	0.92
G(10)	0.44±0.14	0.20
Anova, p=0.07		

Table 7. Expression of *XIST* transcript with gRNAs targeting bases involved pseudoknot formation in the RNA secondary structure.

Number pseudoknot participating bases at the gRNA target (N)	Fold change relative to no insert Mean ±SD	P Value
0	0.34	
1	0.36	0.70
2	0.40	0.72
3	0.38	0.89
Anova, p=0.89		

Table 8. Expression of *SS18-SSX2* transcript and cell death upon treatment with gRNAs at SS and DS regions of the transcript.

gRNA target region (n)	Cell death (%)
Single Stranded (1)	49.3
Double Stranded (1)	12.7
SS-DS Junctions (1)	35.9
Non-target (1)	6.4

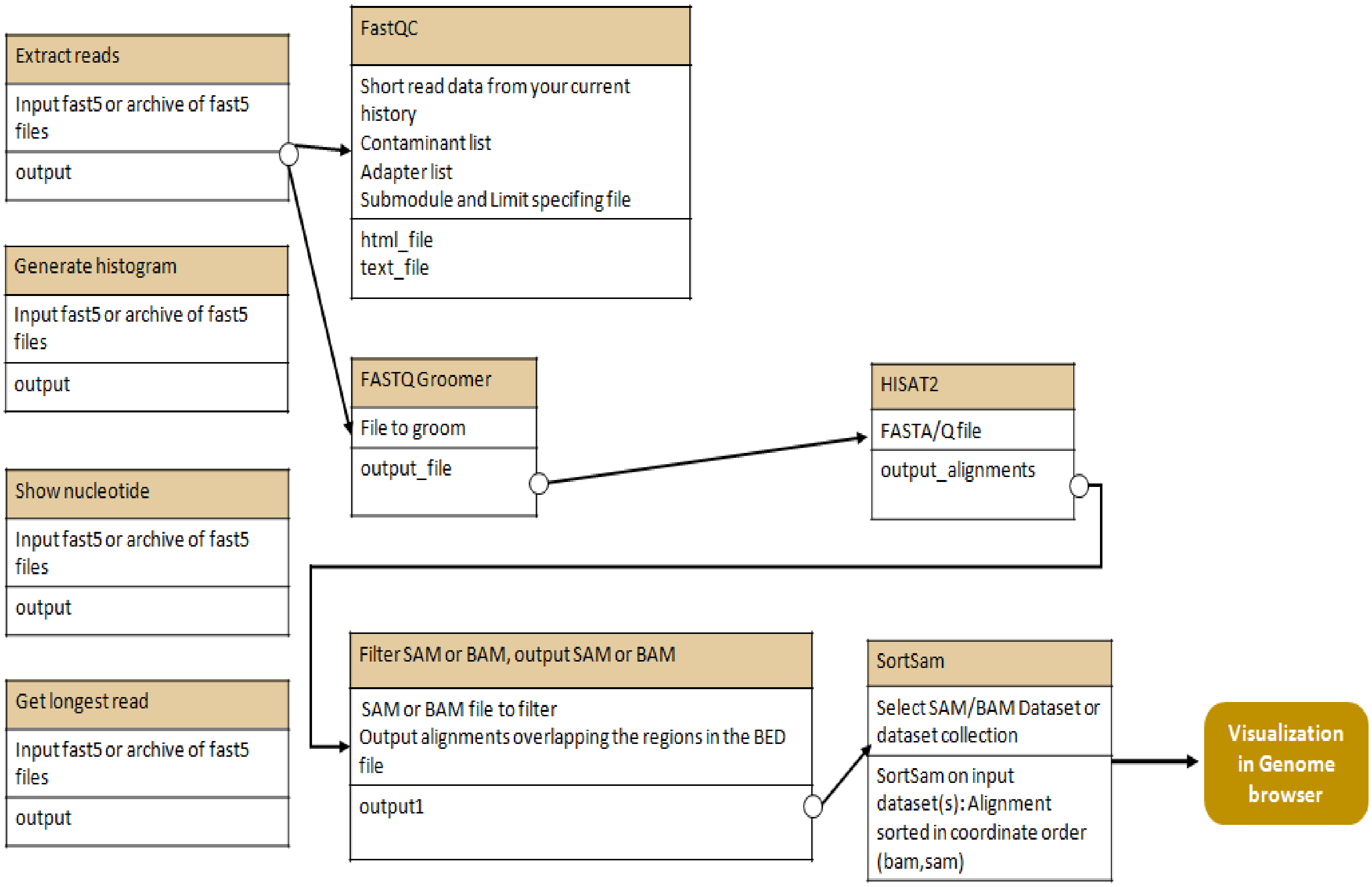

Figure 1. NGS pipeline for Cas13 mediated transcript targeting and cleavage. The complete pipeline was built on Galaxy v 19.01 an open, web-based platform for biological data analysis. Briefly, .fast5 sequence files generated from Nanopore sequencing (MiOxford Nanopore Technologies (ONT), Oxford, UK.) were uploaded in the Galaxy platform. The sequence files were extracted (Extract Reads) for read-lengths (Generate histogram), read-distribution (Show nucleotide) and getting the longest read from the set of .fast5 files (Get longest read). The .fast5 files were parallelly analyzed for the quality of the reads (FastQC) and converted into .fastqsanger format suitable for the downstream mapping programs (FASTQ Groomer). The .fastqsanger files thus generated were mapped onto the Human genome (GRCh38.p12, GRCh38/hg38) by HISAT2 alignment program compatible for RNA-seq. The mapped files generated as .bam and .sam files were further filtered with Map quality Score (MAPQ) (Filter SAM or BAM, output SAM or BAM) to eliminate unmapped reads. The filtered alignment files were further sorted in the order of coordinates (SortSam). The alignments were finally visualized in Integrative Genomics Viewer version 2.5. 2. Finally, cleavage points in the cells treated gRNA-lshCas13 were determined in reference to the cells that lacked plasmid insert.

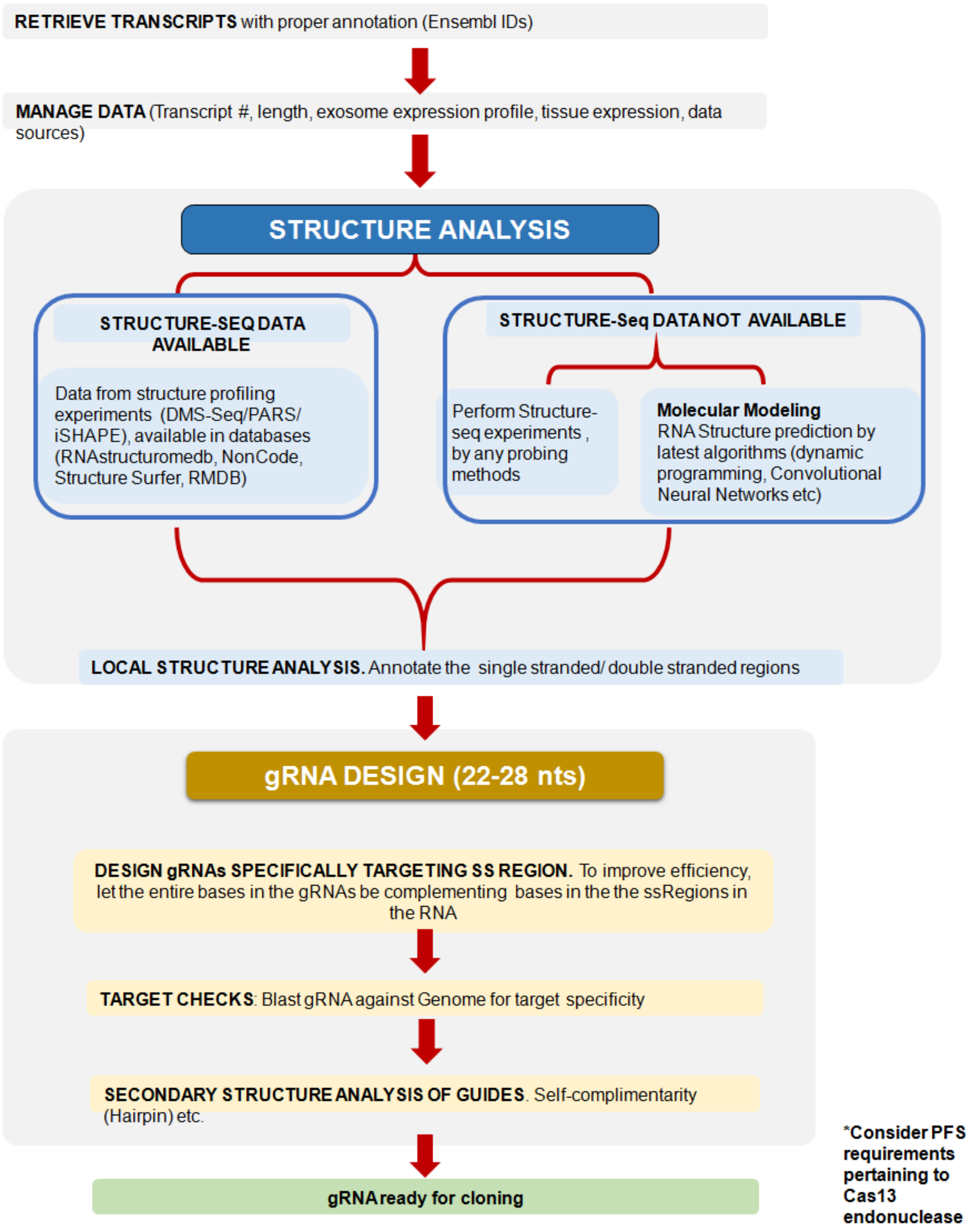

Figure 2. Step-wise workflow for structure based gRNA design for Cas13 mediated RNA targeting and knockdown. The workflow is compatible for structural information derived from structure-seq data as well as from molecular modeling methods. The sole caveat of the strategy lies in designing the gRNAs at the SS regions which will ensure effective targeting of the transcript.
